# A Physiologically Based Pharmacokinetic (PBPK) Study to Assess the Adjuvanticity of Three Peptides in an Oral Vaccine

**DOI:** 10.3390/pharmaceutics16060780

**Published:** 2024-06-08

**Authors:** Leonor Saldanha, Ülo Langel, Nuno Vale

**Affiliations:** 1 PerMed Research Group, Center for Health Technology and Services Research (CINTESIS), Rua Doutor Plácido da Costa, 4200-450 Porto, Portugal; leonorpessanha@gmail.com; 2 CINTESIS@RISE, Faculty of Medicine, University of Porto, Alameda Professor Hernâni Monteiro, 4200-319 Porto, Portugal; 3 Institute of Technology, University of Tartu, Nooruse 1, 50411 Tartu, Estonia; ulo.langel@dbb.su.se; 4 Department of Biochemistry and Biophysics, Stockholm University, 10691 Stockholm, Sweden; 5 Department of Community Medicine, Health Information and Decision (MEDCIDS), Faculty of Medicine, University of Porto, Rua Doutor Plácido da Costa, 4200-450 Porto, Portugal

**Keywords:** vaccines, PBPK, simulation, oral, adjuvant, absorption, peptides

## Abstract

Following up on the first PBPK model for an oral vaccine built for alpha-tocopherol, three peptides are explored in this article to verify if they could support an oral vaccine formulation as adjuvants using the same PBPK modeling approach. A literature review was conducted to verify what peptides have been used as adjuvants in the last decades, and it was noticed that MDP derivatives have been used, with one of them even being commercially approved and used as an adjuvant when administered intravenously in oncology. The aim of this study was to build optimized models for three MDP peptides (MDP itself, MTP-PE, and murabutide) and to verify if they could act as adjuvants for an oral vaccine. Challenges faced by peptides in an oral delivery system are taken into consideration, and improvements to the formulations to achieve better results are described in a step-wise approach to reach the most-optimized model. Once simulations are performed, results are compared to determine what would be the best peptide to support as an oral adjuvant. According to our results, MTP-PE, the currently approved and commercialized peptide, could have potential to be incorporated into an oral formulation. It would be interesting to proceed with further in vivo experiments to determine the behavior of this peptide when administered orally with a proper formulation to overcome the challenges of oral delivery systems.

## 1. Introduction

Adjuvants are used to support the immune response of a vaccine. They are added to the formulation in order to improve their effect [1]. This is possible because they stimulate the innate immune responses and this will also influence the adaptive immune response. Adjuvants are seen as a key element of recent vaccines [2]. When they are added into a vaccine formulation, this allows the reduction in the quantity of antigen, and in certain cases, they increase the stability of the antigen component, extending its half-life and indirectly improving its immunogenic power and the number of vaccination sessions [3,4].

**Scheme 1:**
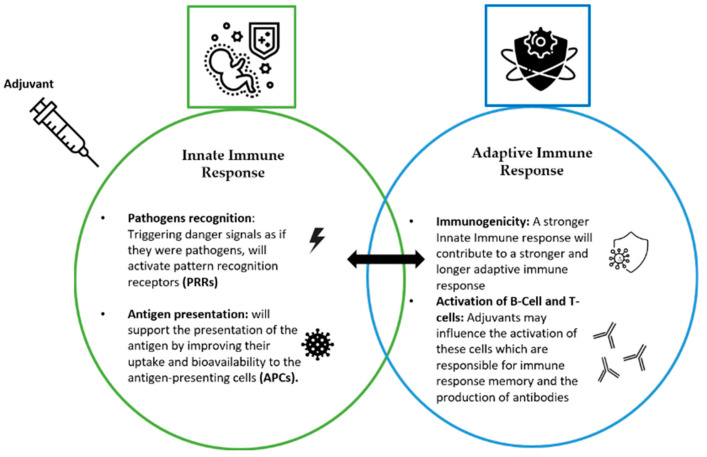
Impact of adjuvants on innate and adaptative immune system.

Based on their mechanism of action, antigens may be classified into delivery systems or immunostimulants [4,5]. Antigens that fall into the category of delivery are composed of components that will support the presentation of the antigen (these can be microparticles or emulsions). The immunostimulants are usually obtained from pathogens (i.e., CpG DNA and lipopolysaccharides), simulating the size and composition of pathogens [6]. They act by providing danger signals that lead to the development and activation of antigen-presenting cells (APCs), as these will target toll-like receptors (TLRs) and other pattern recognition receptors (PRRs). This will enhance the development of antigen signals, which will trigger adaptive immune responses. Scheme 1 explains how immunostimulant adjuvants may interfere with the innate and adaptive immune systems [5,7]. 

It is important to notice that there are not many adjuvants approved for human vaccines. Most formulations have been developed and have been under assessment in clinical trials. Table 1 demonstrates what adjuvants have been licensed and what adjuvants are still under development [3]. Many other adjuvants are still in the pre-clinical stage [8]. This is because not only the efficacy of the adjuvants are important but it is also important to assess their safety [9].

This article will explore how muramyl dipeptides (MDP) and two of its derivates could behavior in an oral formulation. The main goal is to assess if Physiologically Based Pharmacokinetic (PBPK) studies may support the selection of the best adjuvant to be incorporated in an oral vaccine formulation.

In the era of technology, with all the progress that has been made in computer science, in silico studies have become a well-recognized methodology playing an important role in the drug discovery and development process.

In silico is taken from a computer component named silicium. When these kinds of studies are referred to, it means computational approaches are used in their modeling and predictions [10].

They have numerous advantages by reducing time and costs while also impacting the sustainability, since they will avoid the use of animal models leading to more conscious research [11]. There are expectations that in the future, human trials could be replaced at some level by in silico trials, which are able to predict the behavior of a compound in the human body [12]. This is indeed already starting to happen. EMA and FDA are both endorsing these studies and have been providing webinars and guidance to support the drug developers in conducting these studies. For instance, PBPK modeling studies are being used to gather data on drug interactions with another drugs, pediatric population, pregnancy and personalized medicine [13,14].

A PBPK analysis combines physiology, population, and drug characteristics to describe the pharmacokinetic behaviors of a drug. Over the years, the number of articles involving PBPK models has increased, reflecting that this field is maturing and that there is growing interest from many stakeholders, including pharmaceutical industries, health agencies, and academia, in their implementation. These studies are now playing an important role in research and development, post-marketing research, and drug optimization [15,16]. These models can simulate the Absorption, Distribution, Metabolism, and Elimination (ADME) processes and describe drug disposition within the tissues. They are often used to assess plasma-tissue concentrations of the drug over time. Additionally, it is possible to include physiological characteristics in the model, allowing the prediction of pharmacokinetics (PK) and dose under specific disease states or in special populations such as the elderly, children, and pregnant women. These studies also provide capabilities to explore Drug–Drug Interactions (DDIs) and different formulations, allowing predictions of drug behavior if administered through different routes [17,18].

A PBPK analysis combines physiology, population, and drug characteristics. According to this, it is important to acknowledge that these studies are essential for drug discovery as they allow a wide variety of predictions that will support the drug design and the design of clinical trials, saving costs and time. 

In a recent literature review, it was found that there are very few PBPK models and studies for vaccines [19]. Only one PBPK Model with alpha-tocopherol administered intramuscularly was found. The aim of this model was to predict the biodistribution of α-tocopherol in a healthy male human after a single dose of squalene-containing adjuvant through the IM route [20]. For vaccines, in addition to PBPK studies, there are other in silico studies that may help testing immunologic properties and correlates of protection [17]. For instance, a Population Pharmacokinetics (PopPK) model was built in 2021 for an HIV vaccine. The model was used to predict concentrations of an individual-specific HIV-neutralizing antibody under development (VRC01) over time as the first step in the correlates of protection (CoP) in order to analyze the relationship between VRC01 concentration and the instantaneous rate of HIV infection. This information is important to support the development of an HIV vaccine [15].

This evoked the need to conduct research in this area and verify how PBPK studies could support the development of a vaccine formulation administered orally. In this study, PBPK simulations will predict the pharmacokinetic behavior of these peptides when administered orally, and it will be assessed if these peptides have the conditions to overcome the barriers and challenges of an oral formulation to reach their targets in the intestine. Most of these challenges are related to the pH differences across the gastrointestinal tract, physiological barriers, enzymatic activities, and so on [8,21]. 

MDP has not been approved due to its pyrogenic activity [22,23]. However, MDP derivates have been developed in order to diminish MDP side effects and to improve its efficacy. Some of these passed clinical trials and are now licensed as Mepact, the commercial name of mifamurtide (muramyl tripeptide phosphatidyl ethanolamine, MTP-PE) [24]. MDP is the smallest immunostimulant fragment in the cell walls of Gram (+) and Gram (−) bacteria. It is a synthetic derivative of muramyl dipeptide (MDP) with a lipophilic nature [25,26]. Mepact, MTP-PE, has identical immunostimulatory properties as MDP [27]. The addition of alanine and dipalmitoyl phosphatidyl ethanolamine to MDP generates MTP-PE [28].

MTP-PE and MDP have the same activating properties of monocytes and macrophages, being a ligand of the NOD2 receptor which is found in these cells [27]. However, there is a superiority in MTP-PE activation of monocytes due to its lipophilicity which lead to more uptake of cells through passive transfer. MTP-PE is also less toxic [28]. 

Mepact uses liposomes to encapsule MTP-PE to be easily incorporated in the liposomes which are phospholipid vesicles that can be easily captured and by phagocytic cells by monocytes and macrophages located in the liver, spleen, and lungs when administered systemically. Liposomes are powerful drug carriers when administered through injections [25,28].

Considering the crucial role of adjuvants in boosting the immune responses of vaccines, it is important to find new tools and methods capable of supporting the research and development of the right adjuvant and how it can fit into a vaccine formulation. 

This study aims to explore the pharmacokinetic behavior of three peptides when used as adjuvants for an oral vaccine, using the Physiologically Based Pharmacokinetic (PBPK) model developed in the previous research, which created the first PBPK model for an oral vaccine using alpha-tocopherol as an adjuvant [29]. The aim of this research is to assess if these peptides could be considered good adjuvants for an oral vaccine.

## 2. Materials and Methods

### 2.1. Research Literature

The research literature was divided into 3 steps. During Step 1, an exhaustive literature research was conducted using PubMed in order to verify the existing peptides already studied in clinical trials and to assess if there are peptides currently approved being used as adjuvants. After conducting the research, during Step 2, the authors proceeded with the assessment and screening of the articles. Following the screening step, during Step 3, peptides that were found to have clinical studies in place were selected to conduct the Physiologically Based Pharmacokinetic (PBPK) study since when using clinical data, the simulations are more realistic and the safety side of the compound is already studied which may speed up the research process when considering if these peptides could be used to support a vaccine formulation for oral delivery.

#### 2.1.1. Step 1—Research Literature

The construct used to perform the literature research can be seen in Table 2 below.

Since the main goal of this literature research was to verify all existing peptides so far used as adjuvants, all articles found under the above construct were considered and accepted, regardless of the year of publication. 

#### 2.1.2. Step 2—Screening

As exclusion criteria, all articles referring to peptide vaccines instead of peptides used as adjuvants were excluded.

After excluding the out-of-scope articles, a table was built in order to list all peptides that have been studied as vaccine adjuvants, using the following columns:

Peptide Name + Sequence + Vaccine indication + Research Phase + Rational + Year + Source.

#### 2.1.3. Step 3—Selection of Peptides for the PBPK Analysis Based on Results

Since PBPK studies are much more realistic when using clinical data, the peptides extracted from the table above were filtered and only peptides with clinical studies were considered.

It was noticed that many articles focused on muramyl dipeptide (MDP) and its derivates. After further analysis, three MDP peptides were selected: MDP itself and two derivates: MTP-PE (Mepact) and Murabutide.

The MEPACT peptide was the selected peptide since it is approved by EMA and therefore clinical data is available.

### 2.2. PBPK Models Preparation

The same human PBPK model built for the alpha-tocopherol oral vaccine, which was based on the model available for the alpha-tocopherol intramuscular (IM) injection, was used in all MDP, MTP-PE, and murabutide simulations [29,30].

On GastroPlus^®^ v9.8.3, there are five tabs displayed: Compound, Gut Physiology-Species, Pharmacokinetics, Simulation, and Graph. The first three tabs, “Compound”, “Gut Physiology-Species”, and “Pharmacokinetics”, are used to build the simulation model.

The definition of the physicochemical properties and formulation properties of the simulation model is completed on the Compound. In Table 3, very important windows appear on the Compound tab: “Dose No.”, “Dissolution No.”, and “Absorption No”. They can appear red or green. According to the software instructions, if the Dose No. and Dissolution No. indicators appear red while the Absorption No. indicator is green, this indicates that solubility (dose number) and particle size (dissolution number) might limit absorption for this drug, but permeability (absorption number) should not.

To ensure the model optimization, it was considered that the three windows described above should appear green in order to validate the model. This means that the optimized model should overcome any issues related to solubility, dissolution, permeability, and absorption. 

#### 2.2.1. MDP—Model Preparation and Optimization

- First Step

Due to a lack of literature data on physico-chemical properties of this compound, the secondary structure was imported into the ADMET Predictor^®^ module of the Software with the standard administration form and dosage assumed by the software (100 mg tablet; 250 mL). “Absorption No” Window from the Compound tab was red and “Dose No” and “Dissolution No” were green. ADMET Predictor^®^ is a module included in the GastroPlus^®^ v9.8.3 Software. It is considered a machine learning tool which allows data analysis, metabolism prediction and drug design [30].

- Second Step

The researchers kept ADMET Predictor^®^ values; changed the dosage form to capsule dose (4 mg since there is no data from the dosage used in clinical. An amount of 4 mg is the same as used in Mifamurtide, and MDP being more pyrogenic, we will keep the same dosage to better compare) and volume to align with the alpha-tocopherol PBPK Model formulation.

- Third Step

**Figure 1:**
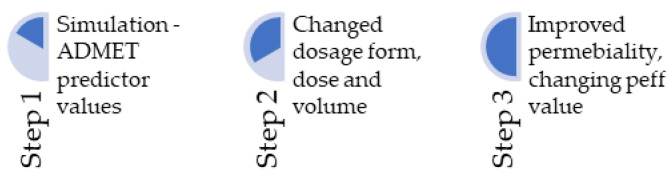
Steps to build optimized MDP model.

As already discussed, absorption in peptides when administered through the oral route are usually compromised by its low logarithm of n-octanol-water partition coefficient (LogP), low permeability in the intestinal mucosa and enzymatic degradation. To overcome these barriers of oral delivery, peptides need to be administered with compounds which are capable of raising the LogP and the permeability and that protect the peptides from enzymatic activities (inhibitors). It is important to acknowledge that in drug research and development, logP works as an indicator of the lipophilicity of the compounds. Lipophilicity plays an important role in the absorption, distribution, metabolism, excretion and toxicity (ADMET) of the drugs. logP will therefore impact the safety and efficacy of the drug being a crucial factor to consider during the drug design [31,32]. Figure 1 summarizes all the steps involved in the preparation of the MDP model.

Therefore, it was assumed that it was necessary to add an excipient or an adjuvant to this formulation in order to improve the oral delivery of MDP. When changing logP values, the absorption did not improve. Considering this, the LogP value predicted by ADMET Predictor^®^ was kept. However, the increase in regional in vivo human intestinal effective permeability (Peff) value increased the absorption [33]. According to the software, the minimum value of Peff that would indicate the best absorption value is a Peff of 1.0. Changing the Peff value was enough to ensure a proper dissolution and absorption of the drug. With this change, the “Absorption No” Window from the Compound tab turned green.

#### 2.2.2. MTP-PE—Model Preparation and Optimization

- First Step

Similarly to MDP, the lack of literature data on the physico-chemical properties of MTP-PE led the researchers to use ADMET Predictor^®^ values. The secondary structure was imported into the ADMET Predictor^®^ module of the software with the standard administration form and dosage assumed by the software (100 mg tablet; 250 mL).

- Second Step

The researchers kept the ADMET Predictor^®^ values and changed the dosage form to a capsule, the dose to 4 mg according to the literature, and the volume to align with the alpha-tocopherol PBPK Model formulation. However, it was found that dissolution and consecutively absorption were very poor, and all compound tab windows were red (“Dose No”, “Absorption No”, and “Dissolution No”).

- Third Step

Considering the same rationale as the MDP model, LogP and Peff values were tested. When changing LogP values, no parameters improved. A Peff value of 1 improved absorption, but there were still issues with the dissolution and bioavailability of the product. The “Absorption No” window turned green.

- Fourth Step

Since MTP-PE is approved by the European Medicines Agency in the form of a liposome (L-MTP-PE) under the name Mifamurtide, and it was found in the literature that the particle size of the liposome is approximately 2–3 μm, this data was added to the system, and all parameters improved slightly. However, the “Dissolution No” window turned green.

- Fifth Step

**Figure 2:**
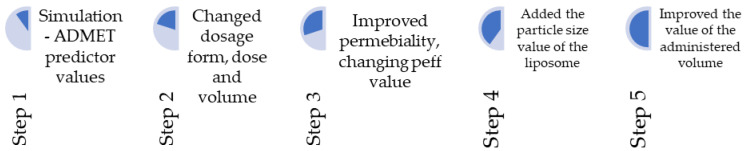
Steps to build optimized MTP-PE model.

In order to improve dissolution, it was determined that a volume of 512 mL had to be administered. This volume was assumed to be ideal for this drug when administered through the oral route to turn the “Dose No” (solubility) window green. Figure 2 summarizes all the steps involved in the preparation of the MTP-PE model.

#### 2.2.3. Murabutide—Model Preparation and Optimization

- First Step

Due to the lack of literature data on the physico-chemical properties of this compound, the secondary structure was imported into the ADMET Predictor^®^ module of the software with the standard administration form and dosage assumed by the software (100 mg tablet; 250 mL). The “Absorption No” window from the Compound tab was red, and the “Dose No” and “Dissolution No” windows were green.

- Second Step

The researchers kept the ADMET Predictor^®^ values and changed the dosage form to a capsule, the dose to 7 mg according to the literature, and the volume to align with the alpha-tocopherol PBPK Model formulation. The “Absorption No” window from the Compound tab was red, and the “Dose No” and “Dissolution No” windows were green.

- Third Step

**Figure 3:**
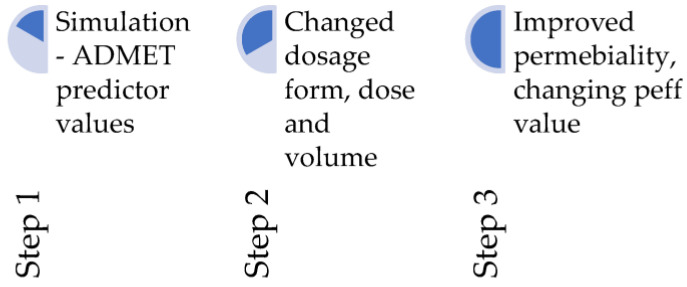
Steps to build optimized murabutide model.

Similarly to MDP and MTP-PE, logP changes did not impact absorption. However, increasing the Peff value improved absorption. According to the software, the minimum Peff value that would indicate the best absorption is 0.6. Changing the Peff value was enough to ensure proper dissolution and absorption of the drug. The “Absorption No” window turned green. Figure 3 summarizes all the steps involved in the preparation of the Murabutide model.

## 3. Results

Simulations were run for the three selected peptides: MDP, MTP-PE and murabutide. Initial simulation results were critical to optimize the models. After having the model optimized, the results from each peptide-optimized model were compared.

### 3.1. Results Related to Models Optimization

- *MDP*

**Figure 4:**
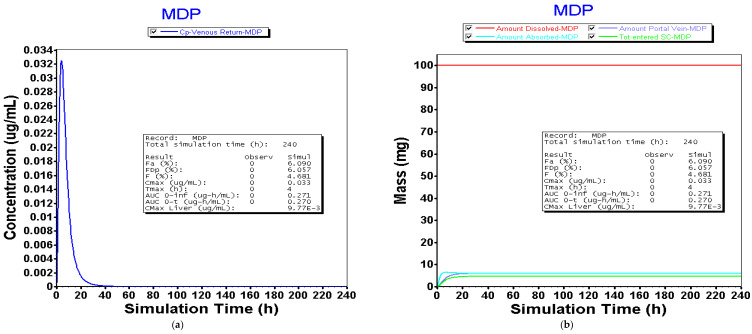

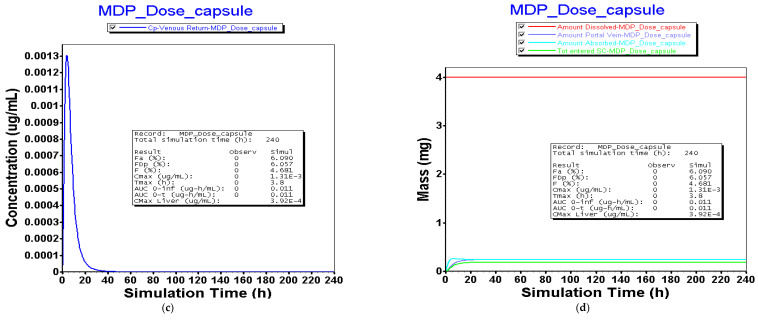

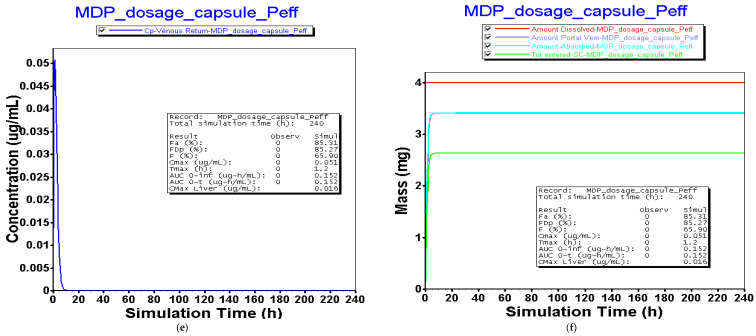
MDP simulation results for the model optimization. (**a**) Plasma concentration without experimental data (ADMET predictions only); (**b**) Absorption and dissolution profile without experimental data (ADMET predictions only); (**c**) Plasma concentration with capsule on dosage form, dose 4 mg and volume 200 mL; (**d**) Absorption and dissolution profile with capsule on dosage form, dose 4 mg and volume 200 mL, (**e**) Plasma concentration with capsule on dosage form, dose 4 mg and volume 200 mL and Peff 1.0; (**f**) Absorption and dissolution profile with capsule on dosage form, dose 4 mg and volume 200 mL and Peff 1.0.

Figure 4a,b assumed ADMET predictor values for a 100 mg tablet. Since the formulation of alpha-tocopherol is integrated in a capsule, this parameter and the volume were changed accordingly. Cmax was 1.31 × 10^−3^ ug/mL according to Figure 4c. On Figure 4b, it is possible to see that the dose of 4 mg was entirely dissolved. However, according to the green and blue lines, this amount of drug is not being metabolized (low amount of dosage in Portal Vein), is not being absorbed and has not entered systemic circulation (green line). This was caused by the poor permeability of the compound. Therefore, the effective permeability value (peff) was improved by assuming that an enhancer of the permeability was added to this formulation, and it was possible to verify in Figure 4e that Cmax improved to 0.05 mL. On Figure 4d, it is possible to see that metabolism and absorption already occurred. Results from Figure 4d,e will be the ones used to compare the behavior of MDP with their derivates (MDP-PE and murabutide).

- *MTP-PE*

**Figure 5:**
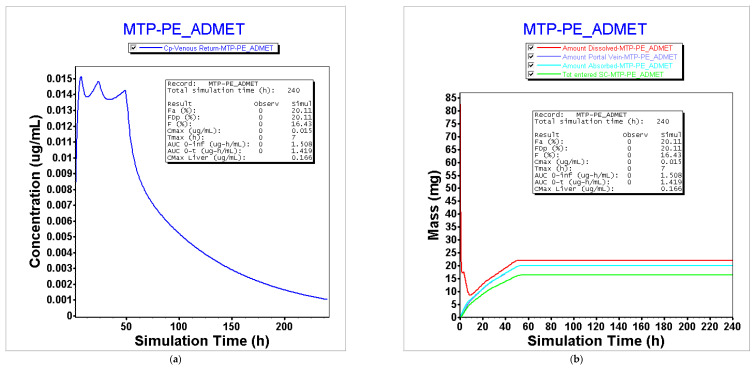

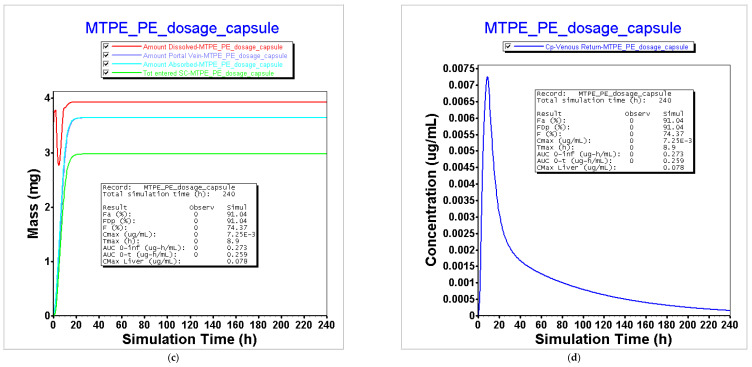

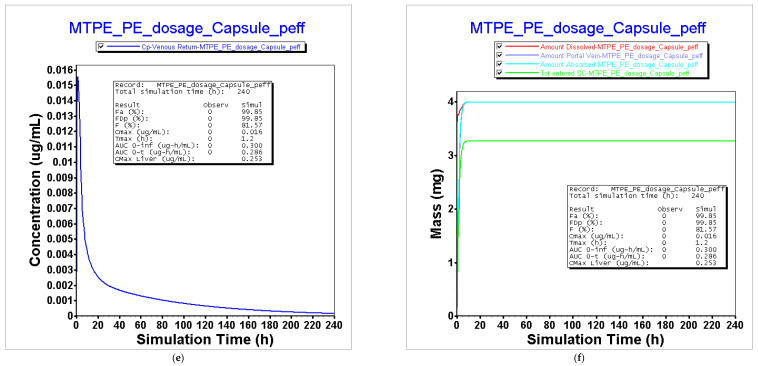

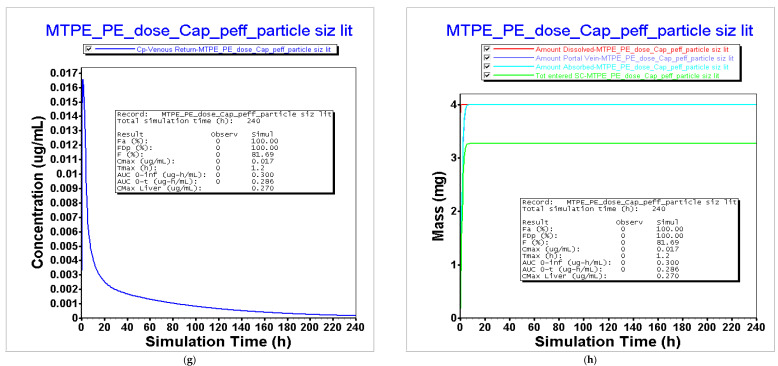

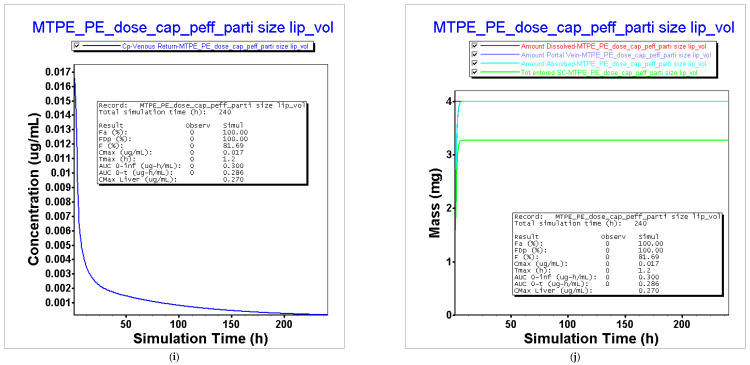
MTP-PE simulation results for the model optimization. (**a**) Plasma concentration without experimental data (ADMET predictions only); (**b**) Absorption and dissolution profile without experimental data (ADMET predictions only); (**c**) Plasma concentration with capsule on dosage form, dose 4 mg and volume 200 mL; (**d**) Absorption and dissolution profile with capsule on dosage form, dose 4 mg and volume 200 mL, (**e**) Plasma concentration with capsule on dosage form, dose 4 mg and volume 200 mL and Peff 1.0; (**f**) Absorption and dissolution profile with capsule on dosage form, dose 4 mg and volume 200 mL and Peff 1.0, (**g**) Plasma concentration with capsule on dosage form, dose 4 mg and volume 200 mL, Peff 1.0; and particle size of 2 μm (**h**) Absorption and dissolution profile with capsule on dosage form, dose 4 mg and volume 200 mL and Peff 1.0 and particle size of 2 μm. And volume of 512 mL, (**i**) Plasma concentration with capsule on dosage form, dose 4 mg and volume 200 mL, Peff 1.0; and particle size of 2 μm (**j**) Absorption and dissolution profile with capsule on dosage form, dose 4 mg and volume 200 mL and Peff 1.0 and particle size of 2 μm and volume of 512 mL.

Figure 5a,b demonstrate the results based on ADMET predictions with its standard assumption of a 100 mg tablet. Since our formulation would be a 4 mg capsule (dosage based on the current commercialized MTP-PE) on the Figure 5c,d it is possible to see the results with these updates. It is possible to see that over time there is dissolution, but Cmax is very low and absorption and systemic circulation are lower than the amount of drug absorbed. Therefore, we improved the Peff in order to verify if these parameters could be enhanced.

In Figure 5e,f, it is possible to verify that absorption increased as indicated by the blue line on Figure 5f and the Cmax also increased. Since MEPACT, the commercialized product of the MTP_PE is a liposome (L-MTP-PE) it was possible to find in the literature its particle size and this was included in the software. Some parameters slightly improved such as Cmax. Although there was not a great difference between Figure 5e or Figure 5f and Figure 5g or Figure 5h since there were small improvements, as the liposome is the commercialized version, we kept the liposome particle size in our model.

Gastroplus^®^ v9.8.3 reported on the Compound tab that Dissolution No. indicators turned green after adding the liposome particle size, which means particle size is not affecting absorption anymore. The Absorption No. was green (meaning permeability was fine which makes sense since we improved the Peff) while the solubility number was red, meaning that dose and volume were impacting the absorption. In order to overcome this and validate our model in the software, we increased the volume. A total of 512 mL is the necessary volume according to Gastroplus^®^ v9.8.3 to ensure a proper absorption of the peptide. Figure 5g–j do not show many differences between themselves. However, we assumed that having all parameters in the compound tab of the program in green is what is required to have a proper model. Considering this, the particle size of the liposome and the volume of 512 mL were selected as the most-optimized model of the MTP-PE peptide that will be used to be compared with the MDP and the murabutide-optimized Models.

- *Murabutide*

**Figure 6:**
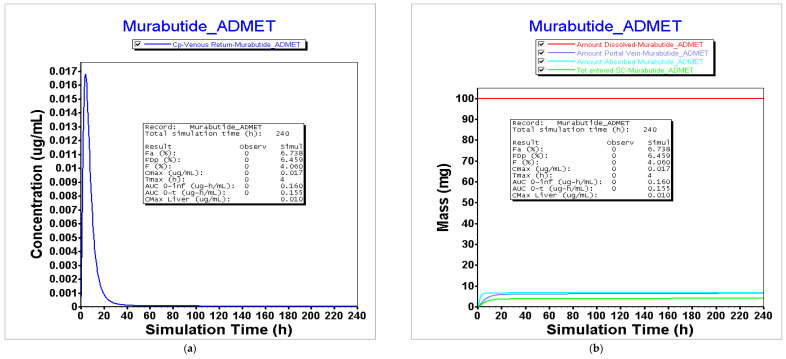

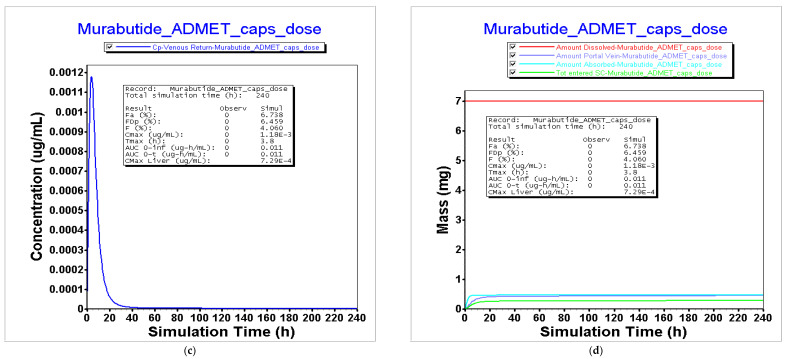

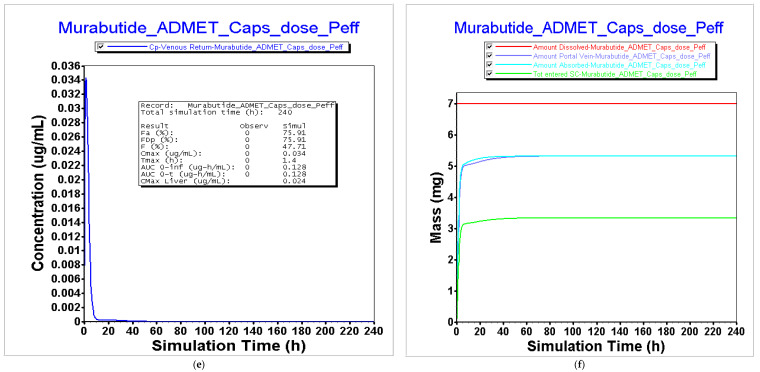
Murabutide simulation results for the model optimization. (**a**) Plasma concentration without experimental data (ADMET predictions only); (**b**) Absorption and dissolution profile without experimental data (ADMET predictions only); (**c**) Plasma concentration with capsule on dosage form, dose 7 mg and volume 200 mL; (**d**) Absorption and dissolution profile with capsule on dosage form, dose 7 g and volume 200 mL, (**e**) Plasma concentration with capsule on dosage form, dose 7 mg and volume 200 mL and Peff 0.6 (**f**) Absorption and dissolution profile with capsule on dosage form, dose 7 mg and volume 200 mL and Peff 0.6.

Figure 6a,b represents the standard predictions from the software. In Figure 6c,d the dosage form was changed to capsules and 4 mg. As can be seen, the absorption and systemic circulation were still extremely poor after these changes. After improving the Peff, we could see an improvement in the absorption Figure 6e,f. All parameters in the compound tab were green, which meant the model was finally optimized. The capsule formulation with a dosage of 7 mg and a Peff of 0.6 was used to compare with the optimized models of MPD and MTP-PE.

### 3.2. Compartmental Absorption Chart Bars of MDP, MTP_PE and Murabutide

- MDP

**Figure 7:**
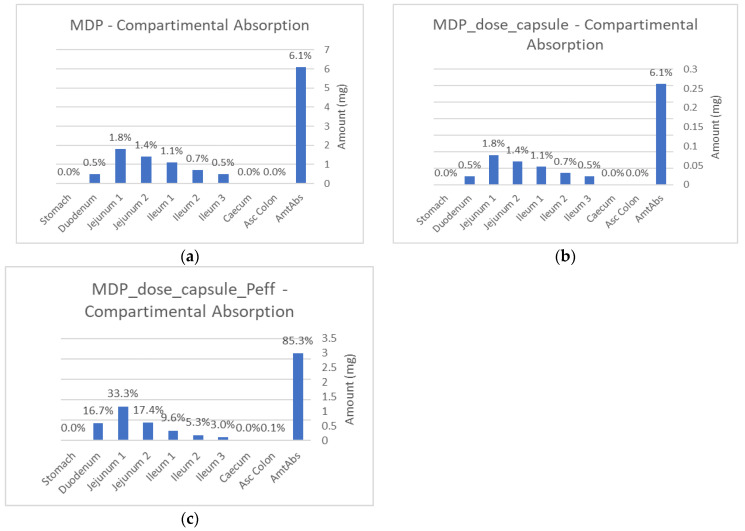
(**a**) Compartmental absorption without experimental data (ADMET predictor only); (**b**) Compartmental absorption with capsule on dosage form, dose 4 mg and volume 200 mL (**c**) Compartmental absorption with capsule on dosage form, dose 4 mg and volume 200 mL and Peff of 1.0.

Figure 7a–c show the compartmental absorption of the drug. When Peff value was improved as shown in Figure 7c, there was a huge increase in the absorption.

- *MTP-PE*

**Figure 8:**
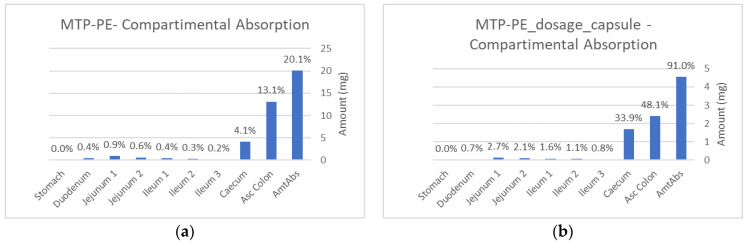

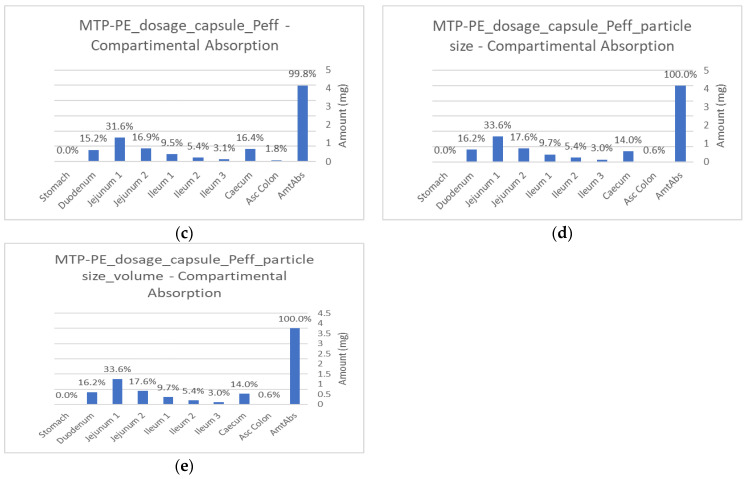
MTP-PE (**a**) Compartmental absorption without experimental data (ADMET predictor only); (**b**) Compartmental absorption with capsule on dosage form, dose 4 mg and volume 200 mL (**c**) Compartmental absorption with capsule on dosage form, dose 4 mg and volume 200 mL and Peff of 1.0 (**d**) Compartmental absorption with capsule on dosage form, dose 4 mg and volume 200 mL and Peff of 1.0 and particle size of 2 μm (**e**) Compartmental absorption with capsule on dosage form, dose 4 mg and volume 200 mL.

Figure 8a–e represent the compartmental absorption chart bar of MTP-PE during model optimization.

- *Murabutide*

**Figure 9:**
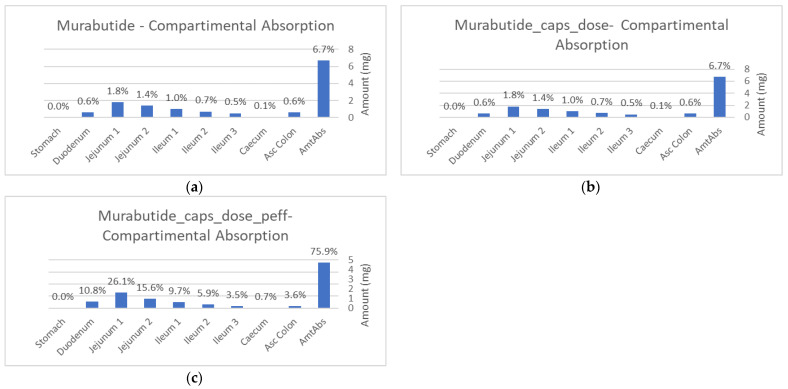
Murabutide (**a**) Compartmental absorption without experimental data (ADMET predictor only); (**b**) Compartmental absorption with capsule on dosage form, dose 7 mg and volume 200 mL (**c**) Compartmental absorption with capsule on dosage form, dose 7 mg and volume 200 mL and Peff 0.6.

Figure 9a–c represent the absorption chart bar for murabutide. It can be seen that after improving Peff (Figure 9c), the absorption increased significantly.

### 3.3. Optimized-MDP vs. Optimized-MTP-PE vs. Optimized-Murabutide

Table 3 compares the obtained pharmacokinetic values of the three optimized models of the peptides, MDP, MTP-PE, murabutide and alpha-tocopherol, after running the simulations.

## 4. Discussion

### 4.1. Summary of Findings

It was found in the literature that one of the most limiting factors that is blocking the use of peptides in oral vaccines is their poor permeability [34,35]. In our study, the main important finding related to the optimization of our models and oral vaccine formulations of the three peptides was aligned with the literature since a low permeability was seen in all the compounds and absorption increased after improving the Peff value.

According to the Biopharmaceutical Classification System, Peff, which represents the human effective intestinal membrane permeability, is one of the main factors that play an important role in drug absorption [36]. Therefore, in order to be possible to use peptides in oral formulations, a permeation enhancer would be required. This is also aligned with the information found in the literature [37].

Table 2 compares the pharmacokinetic values between the optimized models for MDP, MTP-PE and murabutide against the alpha-tocopherol. It is possible to see that in terms of Tmax, all peptides are very similar to each other and also with alpha-tocopherol when administered orally. AUC is higher in the optimized-MTP-PE model when compared with the other MDP peptides and this is related to the higher bioavailability being higher than the other peptides. Optimized MTP-PE has a similar bioavailability to alpha-tocopherol. For these reasons, and considering also that this peptide is already approved but in other pharmaceutical form and used as an adjuvant by EMA, the researchers believe that MTP-PE would be the best peptide to use in an oral formulation for a vaccine.

### 4.2. Context of Findings

MDP is not being clinically used due to its pyrogenicity. Only its derivates are used. In this study, MDP derivates such as mifamurtide and murabutide that are currently used as adjuvants through other administration routes are assessed using in silico studies to determine if they could be used as adjuvants administered through oral route alone or together with alpha-tocopherol, using the same PBPK model that was built for the latest. 

Mifarmurtide is being used with a dosage of 4 mg and murabutide with a dosage of 7 mg. For MDP, we assumed the 4 mg dosage since due to its pyrogenicity, it would be better to keep the same dosage used for Mifarmurtide.

As described in the results section, it was found that MDP has a poor permeability which is impacting absorption and metabolism.

It is reported in the literature that the barriers related to the use of peptides through the oral route are associated with their characteristics such as their large molecular size and poor permeation, metabolism, and degradation in the gastrointestinal tract. In order to overcome these challenges, usually an oral peptide formulation will contain some enzyme inhibitors or substances capable of improving permeation [38]. 

Therefore, for the MDP peptide, it was considered that an enhancer of the permeability was added to this formulation. After running simulations with a higher value of Peff, it was possible to see that Cmax improved and both metabolism and absorption started to show progress. 

The compartmental absorption of the drug also corroborates this. It can be seen that when Peff value was improved the absorption had a huge increase (Figure 7a–c).

For MTP-PE, since the commercialized version is 4 mg, this was the dosage considered for our simulations. As we are simulating through the oral route, for the pharmaceutical form we selected a capsule. For this compound, we also had to improve Peff in order to see an increase in the absorption and we added the particle size of the liposome found in the literature. Some parameters only slightly improved with the addition of the particle size but the main difference is that Dissolution No and Absorption No of the compound tab of the software turned green after adding the liposome particle size meaning that particle size was not affecting absorption and that permeability was fine. However, solubility was still red in the software which meant that dose and volume were impacting our value. Therefore, to overcome this, different volumes were tested, and it was verified we needed to increase it in order to overcome this situation. The optimized model used to compare this compound with MDP and murabutide had a volume of 512 mL and the liposome particle size.

On murabutide, the same rationale was applied. First, we changed the tablet and the dosage from the software on Figure 6a,b to capsules and to the dosage found in the literature (Figure 6c,d). Absorption and systemic circulation were still very poor but increased after improving the Peff, which means permeability was also a limitation of this compound. It was found in the literature clinical Phase 1 and Phase 2 trials for murabutide using 7 mg, leading to a good tolerability with clinical effects observed and with an improvement in the immunity of HIV-infected patients [39]. Considering the above, 7 mg was the dosage selected to run the simulations. Therefore, the capsule formulation with a dosage of 7 mg and a Peff of 0.6 was used to compare with the optimized models of MPD and MTP-PE.

As mentioned earlier, low permeability is reported in the literature as a barrier to peptide development for oral administration.

After analyzing the results from the model optimization process, we will now compare the most-optimized model of the three peptides.

When comparing the results from Table 2, between the optimized models for MDP, MTP-PE and murabutide against the alpha-tocopherol, it can be seen that Tmax of all peptides and alpha-tocopherol are similar. AUC is higher in the optimized-MTP-PE model in comparison with the other MDP peptides. In addition to this, it is known that optimized MTP-PE has a similar bioavailability to alpha-tocopherol. 

Due to the above and the fact that MTP-PE is already commercialized and used as an adjuvant in the oncology field, the researchers believe that it further experiments could explore the use of MTP-PE as an adjuvant for an oral vaccine.

### 4.3. Limitations

For future research, it would be interesting to test in a non-clinical setting an oral formulation of the MTP-PE with a liposome, adding permeation enhancers and an enzyme inhibitor. As previously reported, in the literature it is found that not only the low permeability is an obstacle to the use of peptides in oral formulations but also their enzymatic degradation. Therefore, it is acknowledged that in this article the metabolism is not studied and this is a limitation that would need to be explored to bring more knowledge to the hypothesis of using MTP-PE in an oral formulation. In addition to this, would be interesting to add alpha-tocopherol to this formulation and verify if there is any synergy in terms of immune response when this peptide is administered together with alpha-tocopherol.

## Figures and Tables

**Table 1:** Adjuvants that are licensed and adjuvants that are still in clinical development.

Adjuvant	
Licensed	Clinical Trials
Aluminium salts	Freund’s adjuvant
MF59	Poly-IC
R837/R848	MDP
AS04	CpG ODN
AS03	AS01
	Flagellin
	AS02

**Table 2:** Construct used to perform the literature research.

Construct					
Pubmed	vaccine* (Title)	AND	“peptide*” (Title)	AND	“adjuvant*” (Title)

**Table 3:** Comparison of optimized models for MDP, MTP-PE, murabutide and alpha-tocopherol.

Results			Peptides	Alpha-Tocopherol Oral PBPK Model [29] Capsule 150 mg Volume 200 mL
	Optimized-MDP	Optimized-MTP-PE	Optimized-murabutide	
Fa (%)	85.31	100.0	75.91	100
FDp (%)	85.27	100.0	75.91	100
F (%)	65.90	81.69	47.71	82.096
Cmax (μg/mL)	0.051	0.017	0.034	1.0473
Tmax (h)	1.2	1.2	1.4	0.8
AUC _0-inf (μg-h/mL)_	0.152	0.300	0.128	14.596
AUC _0-t (μg-h/mL)_	0.152	0.286	0.128	12.501
Cmax liver (μg/mL)	0.016	0.270	0.024	19.28

Fa (%)—This shows the net (absorption minus exsorption) percent of the dose that has moved from the lumen into the enterocytes at the time shown above; FDp (%)—Shows the net percent of the dose that has reached the portal vein. In the absence of gut metabolism and drug accumulation in enterocytes, this number will be equal to Fa (%) (for oral doses); F (%)—%Bioavailable. If there is no liver metabolism for this drug, F (%) will be equal to FDp (%) (for oral doses). If the drug does not undergo either one of the processes like gut metabolism, accumulation in gut tissue, or liver metabolism, all three parameters—Fa (%), FDp (%) and F% will be equal; AUC _0-inf (ng-h/mL)_—This shows the current value of the area under the central compartment plasma-concentration–time curve. This value is AUC extrapolated to infinity; AUC _0-t (ng-h/mL)_—This shows the current value of the area under the central compartment plasma-concentration–time curve. This value is AUC for the time of the simulation—it is not extrapolated to infinity.

## Data Availability

Data are contained within the article.
